# Coexistence of Nerve Enlargement and Neuratrophy Detected by Ultrasonography in Leprosy Patients

**DOI:** 10.1038/s41598-018-26085-1

**Published:** 2018-05-17

**Authors:** Xiaohua Chen, Liangfu Zhang, Meiying Huang, Xiuli Zhai, Yan Wen, Chunzhi Pan

**Affiliations:** 1 Beijing Tropical Medicine Research Institute, Beijing Friendship Hospital, Capital Medical University, Beijing, 100050 China; 2 Beijing Key Laboratory for Research on Prevention and Treatment of Tropical Diseases, Capital Medical University, Beijing, 100050 China; 3 Sanya Leprosy Prevention and Control Center, Sanya, Hainan 572000 China; 4 China Leprosy Association, Beijing, 100068 China

## Abstract

The purpose of this study was to evaluate peripheral neural impairment in leprosy patients by ultrasonography (US). The cross-sectional areas (CSAs) of the median (M), ulnar (U) and common fibular (CF) nerves were compared in 71 leprosy patients and 29 healthy controls, and the data were analyzed between the leprosy, multibacillary (MB)/paucibacillary (PB), reaction (R)/no reaction (NR), disability (D)/no disability (ND), and longer/shorter duration groups after treatment. We found that for the nerves located in upper limbs, the CSAs were significantly increased in the leprosy patients vs the controls; the PB group vs the MB group; the R group vs the NR group; the ND group vs the D group; and the longer duration group vs the shorter duration group at some positions of the M nerve and U nerve. In contrast, for the nerves located in lower limbs, the CSAs were significantly reduced in the leprosy patients vs the controls and in the longer duration group vs the shorter duration group at some positions of the CF nerve. This result indicated that nerve enlargement and neuratrophy coexist in leprosy patients.

## Introduction

Leprosy is an infectious disease caused by *Mycobacterium leprae* that predominantly affects the skin and peripheral nerves^1,2^. The acute and chronic neurological involvement have been increasingly recognized as major components of this infection^3^. The resulting nerve damage affects the sensory, motor and autonomic systems and characteristically results in nerve enlargement^4^, which may start before diagnosis or either during or after treatment, leading to functional impairments and deformities^4–6^.

Early diagnosis of nerve damage in leprosy patients is possible using conduction studies, thermal perception testing and nerve palpation (to detect thickening), but these techniques are examiner dependent and require practical training^6^. Studies have reported that clinical examination of enlarged nerves in leprosy patients is subjective and inaccurate, whereas ultrasonography (US) provides an objective measure of nerve damage by providing information on the location and degree of nerve enlargement, nerve morphologic alterations, and echo texture, fascicular pattern, and vascularity of the nerve^7–11^. These features reflect nerve histologic changes and are useful for the study of structural changes in nerve sites that cannot be biopsied for histopathologic examination^9^. In addition, nerve US is a portable, noninvasive, easily available and cost-effective tool that has the potential to become the primary modality for the evaluation of focal peripheral nerve disorders^8,9,12^.

The median (M), ulnar (U) (at the cubital tunnel (Ut) and proximal to the tunnel (Upt)) and common fibular (CF) nerves represent the most critical peripheral nerves of leprosy patients assessed by US. Comparisons of cross-sectional areas (CSAs) between leprosy patients and controls have revealed extensive enlargement of the U, M and CF nerves^10,13–15^. Of these nerves, extensive enlargement of the U nerve, as assessed by US, is the characteristic sign of neuropathy in leprosy patients^10,13^. One study reported that the CSAs of nerves were thicker in multibacillary (MB) patients than those in paucibacillary (PB) patients, regardless of whether the readings were obtained before or after multi-drug therapy (MDT). For PB patients, the CF nerve (left) exhibited significant enlargement after MDT compared to that before MDT (p < 0.01). In contrast, for MB patients, the U (Ut (left and right) and Upt (left)) and M (left) nerves were significantly thinner (p = 0.02, 0.04, p = 0.03 and p = 0.04, respectively)^16^. The CSAs of the nerves in patients with a reaction (R) were thicker than those in patients with no reaction (NR) before and after MDT. Regarding patients with NR, the CF nerve (left) exhibited significant enlargement after MDT compared to before MDT (p = 0.02). In contrast, for patients in the R group, the U nerve (Upt (left)) was significantly thinner (p = 0.03)^16^. Another study compared the CSAs of the nerves in PB and MB patients with or without a reaction before MDT and found that nerve enlargement occurred in the MB and R group. In addition, significant differences in asymmetry indexes, ΔCSA (absolute difference between the right and left CSAs) and ΔUtpt (absolute difference between the Upt and Ut CSAs) were noted in the R group^14^.

The purpose of this study was to investigate peripheral nerve damage in leprosy patients during or after MDT using the CSA according to US measurements at the following positions: the bilateral M (1/2 of the forearm and 1/3 of the forearm), U (Ut and Upt) and CF nerves. Differences in the CSAs were compared between the leprosy patients and the controls, the MB and PB groups, the R and NR groups, the disability (D) and no disability (ND) groups and the different treatment durations (>20 years and <20 years) to assess the influence of clinical characteristics on nerve damage.

## Results

### Basic Characteristics of the Leprosy Patients and the Controls

The average ages ± standard deviation (SD) of the patients and the controls were 61.9 ± 14.5 years and 57.6 ± 17.9 years, respectively. In total, 48 (67.6%) of the leprosy patients were men and 23 (32.4%) were women. Seventeen (58.6%) of the controls were men and 12 (41.4%) were women. The ethnicities of the leprosy patients included Han (22.5%), Miao (2.8%), Li (73.2%) and Hui (1.4%). Leprosy classifications, reaction, disability and duration are presented in Table 1. Basic characteristic of the leprosy patients and the controls enrolled in this study are presented in Table 2.

**Table 1:** Clinical characteristics of the leprosy patients enrolled in this study.

Leprosy classification (n, %)				Leprosy reactions (n, %)			Leprosy disability (n, %)		Leprosy treatment duration (n, %)	
WHO*		RJ**		Type 1	Type 2	NR	ND	D	SD	LD
MB	37(52.1)	LL	23(62.2)	0(0)	3(60)	20(31.7)	11(37.9)	12(28.6)	5(27.8)	18(34.0)
		BL	8(21.6)	0(0)	2(40)	6(9.5)	7(24.1)	1(2.4)	5(27.8)	3(5.7)
		BB	6(16.2)	1(33.3)	0(0)	5(7.9)	5(17.2)	1(2.4)	2(11.1)	4(7.5)
PB	34(47.9)	BT	8(23.5)	1(33.3)	0(0)	7(11.1)	4(13.8)	4(9.5)	5(27.8)	3(5.7)
		TT	26(76.5)	1(33.3)	0(0)	25(39.7)	2(6.9)	24(57.1)	1(5.5)	25(47.1)
Total	71(100)		71(100)	3(100)	5(100)	63(100)	29(100)	42(100)	18(100)	53(100)

n: number of patients, with percentages in parentheses. *WHO: Operational classification proposed by the World Health Organization. **RJ: Ridley-Jopling classification. NR: no reaction; ND: no disability; D: disability.

**Table 2:** Basic characteristic of the leprosy patients and the controls.

Classification		Number of cases	Age (years)	Gender (n, %)		Ethnicities (n, %)			
				Male	Female	Li	Han	Miao	Hui
Leprosy		71	61.9 ± 14.5	48 (67.6)	23 (32.4)	52(73.2)	16(22.5)	2(2.8)	1(1.4)
Controls		29	57.6 ± 17.9	17(58.6)	12 (41.4)	No data	No data	No data	No data
WHO*	MB	34	63.2 ± 14.9	22(64.7)	12(35.3)	26(76.5)	6(17.7)	1(2.9)	1(2.9)
	PB	37	62.6 ± 14.3	26(70.3)	11(29.7)	26(70.3)	10(27.0)	1(2.7)	0(0.0)
Ridley-Jopling**	TT	26	66.2 ± 12.3	18(69.2)	8(30.8)	22(84.6)	2(7.7)	1(3.8)	1(3.8)
	BT	8	49.3 ± 14.4	4(50.0)	4(50.0)	4(50.0)	4(50.0)	0(0.0)	0(0.0)
	BB	6	59 ± 21.5	5(83.3)	1(16.7)	3(50.0)	3(50.0)	0(0.0)	0(0.0)
	BL	8	57 ± 13.6	7(87.5)	1(12.5)	6(75.0)	2(25.0)	0(0.0)	0(0.0)
	LL	23	66.2 ± 11.9	14(60.9)	9(39.1)	17(73.9)	5(21.7)	1(4.3)	0(0.0)
Reaction	R	8	41.6 ± 10.8	7(87.5)	1(12.5)	3(37.5)	5(62.5)	0(0.0)	0(0.0)
	NR	63	65.6 ± 12.6	41(65.1)	22(34.9)	49(77.8)	11(17.5)	2(3.2)	1(1.5)
Disability	D	51	65.5 ± 11.9	34(66.7)	17(33.4)	42(82.4)	6(11.8)	2(3.9)	1(1.9)
	ND	20	56.1 ± 18.2	14(70.0)	6(30.0)	10(50.0)	10(50.0)	0(0.0)	0(0.0)
	Longer	53	68.3 ± 10.6	32(60.4)	21(39.6)	43(81.1)	7(13.2)	2(3.8)	1(1.9)
	Shorter	18	46.9 ± 12.5	16(88.9)	2(11.1)	9(50.0)	9(50.0)	0(0.0)	0(0.0)

n: number of patients, with percentages in parentheses. *WHO: Operational classification proposed by the World Health Organization. **RJ: Ridley-Jopling classification. NR: no reaction; ND: no disability; D:disability; No data: no data collected.

### Nerve CSA Measurement

**Figure 1:**
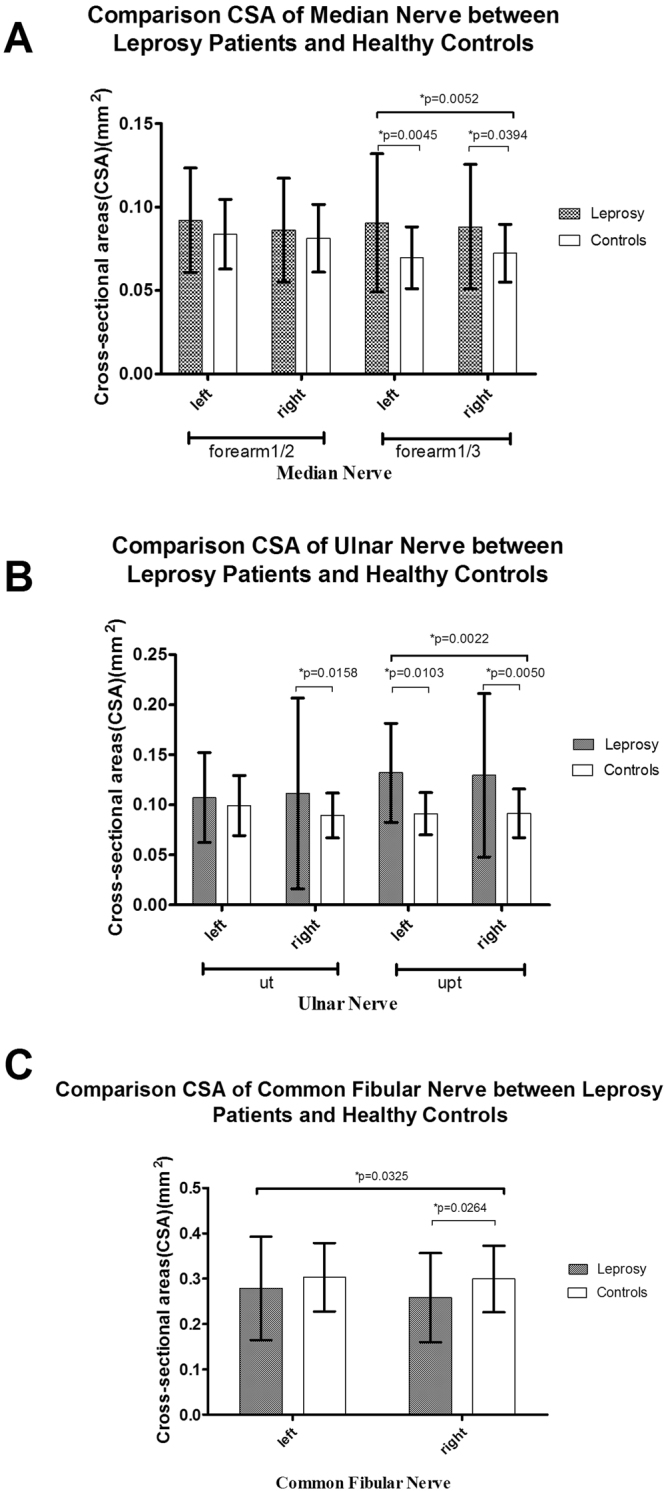
CSA results of the leprosy patients and the healthy controls. (**A**) CSAs of the median (M) nerve at 1/2 and 1/3 of the forearm; (**B**) CSAs of the ulnar (U) nerve at the cubital tunnel area (Ut) and the area proximal to the tunnel (Upt).; and (**C**) CSAs of the common fibular (CF) nerve.

**Figure 2:**
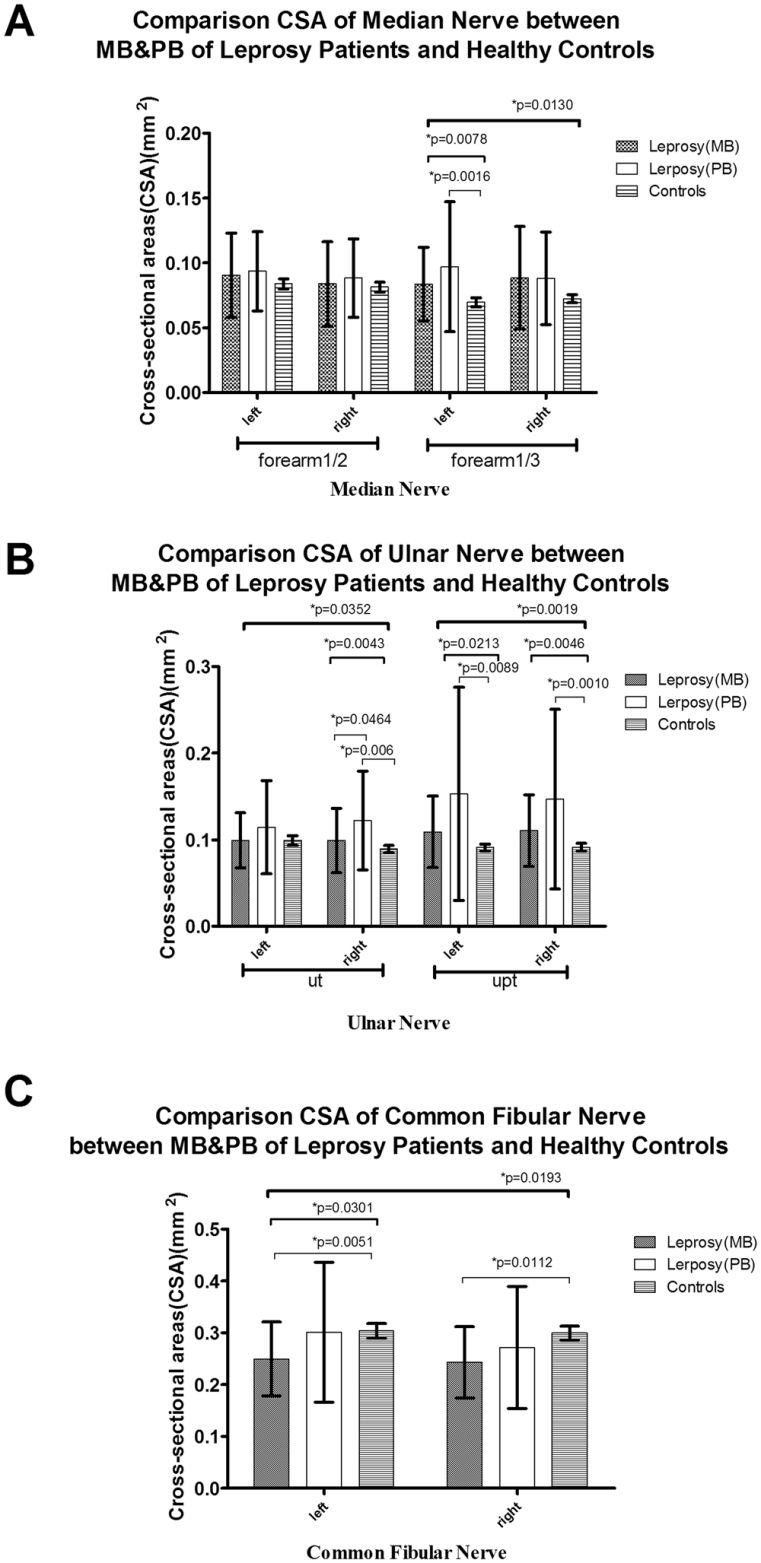
CSA results of the multibacillary (MB) and paucibacillary (PB) groups of leprosy patients and healthy controls. (**A**) CSAs of the median (M) nerve at 1/2 and 1/3 of the forearm; (**B**) CSAs of the ulnar (U) nerve at the cubital tunnel area (Ut) and the area proximal to the tunnel (Upt).; and (**C**) CSAs of the common fibular (CF) nerve.

**Figure 3:**
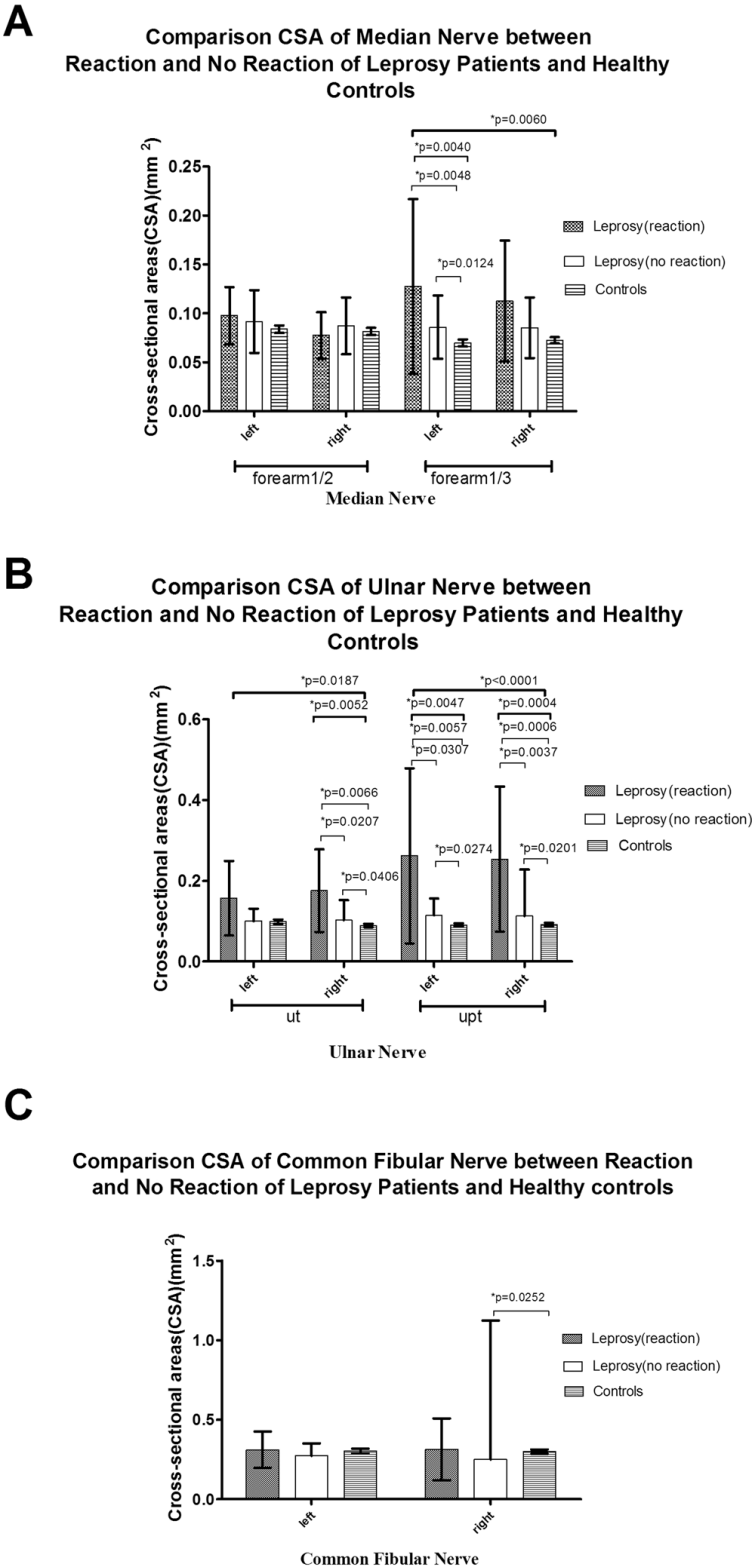
CSA results of the reaction (R) and no reaction (NR) groups of leprosy patients and healthy controls. (**A**) CSAs of the median (M) nerve at 1/2 and 1/3 of the forearm; (**B**) CSAs of the ulnar (U) nerve at the cubital tunnel area (Ut) and the area proximal to the tunnel (Upt).; and (**C**) CSAs of the common fibular (CF) nerve.

**Figure 4:**
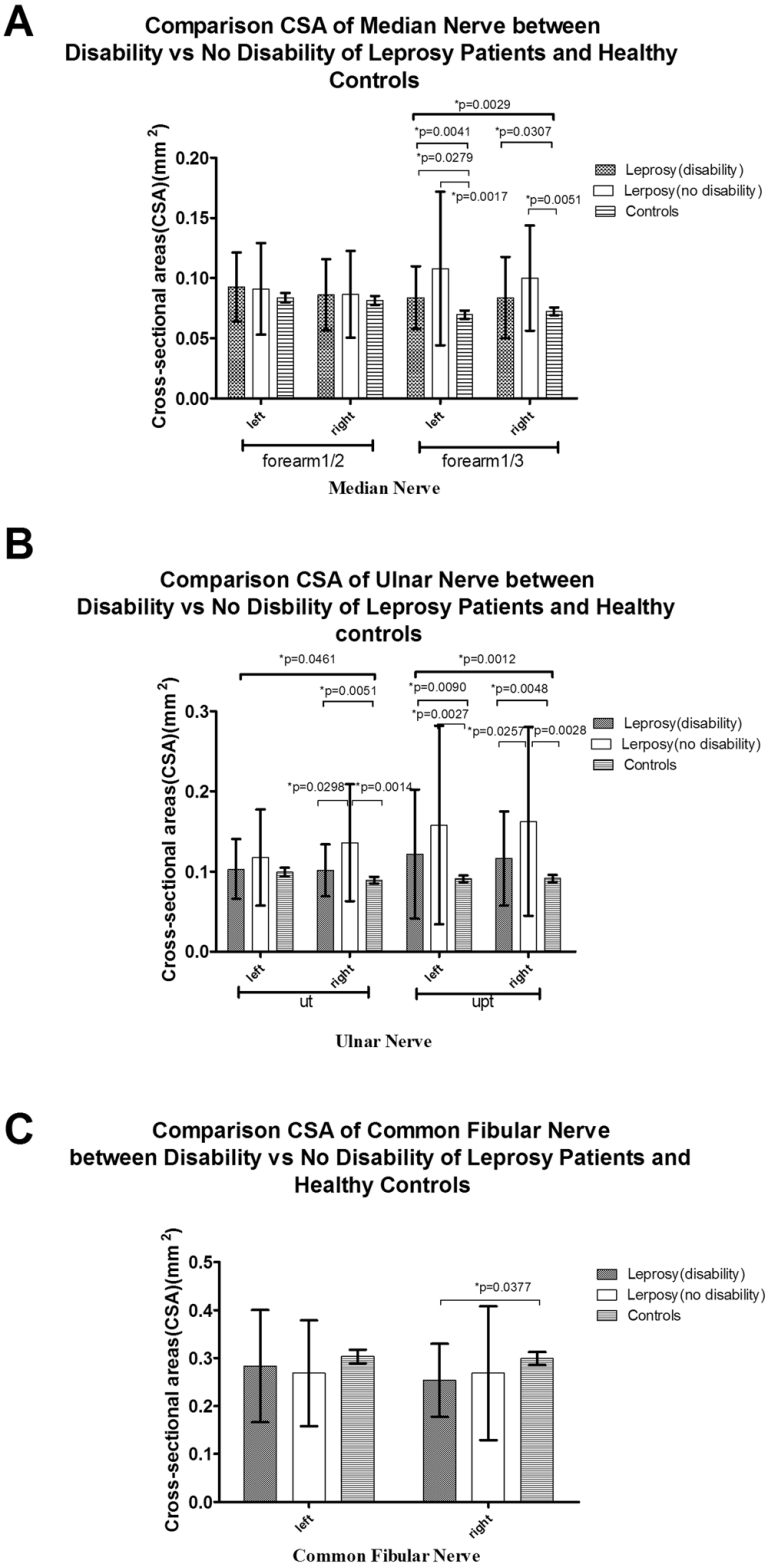
CSA results of the disability (**D**) and no disability (ND) groups of leprosy patients and healthy controls. (**A**) CSAs of the median (M) nerve at 1/2 and 1/3 of the forearm; (**B**) CSAs of the ulnar (U) nerve at the cubital tunnel area (Ut) and the area proximal to the tunnel (Upt).; and (**C**) CSAs of the common fibular (CF) nerve.

**Figure 5:**
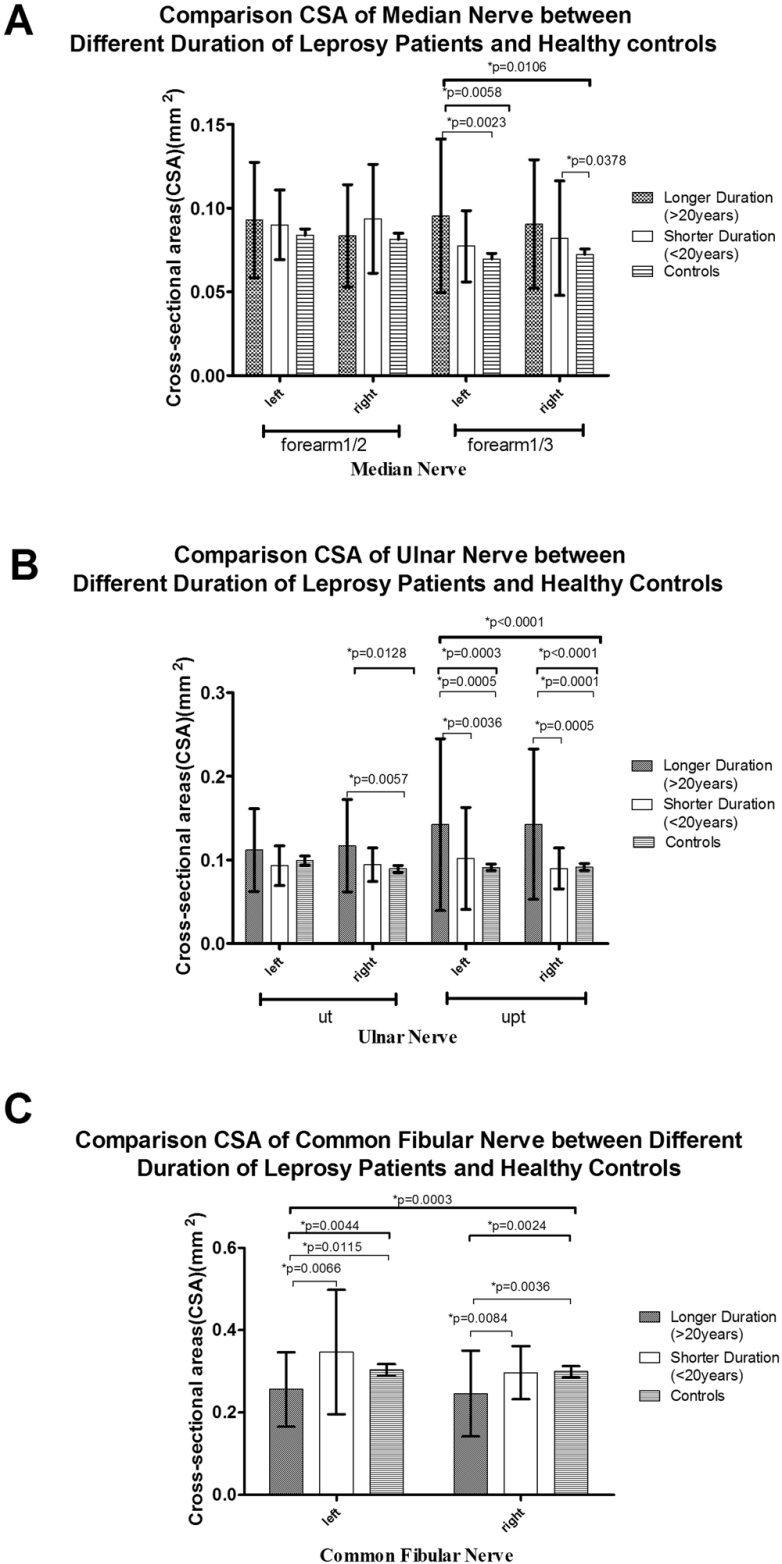
CSA results among the leprosy patients with different treatment durations and the healthy controls. (**A**) CSAs of the median (M) nerve at 1/2 and 1/3 of the forearm; (**B**) CSAs of the ulnar (U) nerve at the cubital tunnel area (Ut) and the area proximal to the tunnel (Upt).; and (**C**) CSAs of the common fibular (CF) nerve.

Figures 1–5 present the CSAs of peripheral nerves in the different groups as the means, SDs, and p-values for differences between the groups. The means, SDs, number of nerves(n), 95% confident interval(CI) and p-values are presented in Supplementary Tables S1,S2. Clinical characteristics were compared among the following groups: leprosy/controls, MB/PB/controls, R/NR/controls, D/ND/controls and the different treatment durations compared to the controls.

The mean ± SD of CSA of the nerves located in upper limbs (M and U nerves) exhibited the following trends: leprosy patients > controls (Fig. 1A,B), PB > MB > controls (Fig. 2A,B), R > NR > controls (Fig. 3A,B), ND > D > controls (Fig. 4A,B), and longer > shorter duration > controls (Fig. 5A,B). However, these trends were not noted at the M nerve (forearm1/2, right): NR < R < controls and shorter > longer duration > controls. In contrast to the CSAs of the nerves located in upper limbs (M and U nerves), which were increased in leprosy groups (Figs 1–5A,B), the CSAs of the nerves located in lower limbs (CF) exhibited the following trends: leprosy patients < controls (Fig. 1C), MB < PB < controls (Fig. 2C), NR < R < controls (Fig. 3C), D < controls and ND < controls (Fig. 4C). The smallest CSA was noted in the longer duration group (Fig. 5C).

For the M nerve, significant differences existed in M (forearm 1/3), but not in the M (forearm 1/2): (1) the leprosy group vs the control group (bilateral, p = 0.0045 left and p = 0.0394 right) (Fig. 1A); (2) the PB group vs the control group (left, p = 0.0016) (Fig. 2A); (3) the R group and the NR group vs the control group (left, p = 0.0048 and p = 0.0124, respectively) (Fig. 3A); (4) the D group vs the control group (left, p = 0.0279) and the ND group vs the control group (bilateral, p = 0.0017 left and p = 0.0051 right) (Fig. 4A); (5) the longer duration group vs the control group (left, p = 0.0023) and the shorter duration group vs the control group (right, p = 0.0378) (Fig. 5A).

For the U nerve, significant differences mainly existed in the U(Upt, bilateral) but also in U (Ut, right): (1) the leprosy and control groups at the U nerve (Ut, right, and Upt, bilateral, p = 0.0158,p = 0103 left and p = 0.0416 right, respectively) (Fig. 1B); (2) the MB and PB groups at the U nerve (Ut, right, p = 0.0464) (Fig. 2B), and the PB and control groups at the U nerve (Ut, right and Upt, bilateral, p = 0.0006, p = 0.0089 left and p = 0.0010 right) (Fig. 2B); (3) the R and NR groups (p = 0.0207, p = 0.0307 and p = 0.0037), the R and control groups (p = 0.0066, p = 0.0057 and p = 0.0006) and the NR and control group (p = 0.0406, p = 0.0274 and p = 0.0201) at the U nerve (Ut, right and Upt, bilateral) (Fig. 3B); (4) the D and ND groups at the U nerve (Ut and Upt, right, p = 0.0298 and p = 0.0257, respectively) (Fig. 4B) and the ND and control groups at the U nerve (Ut, right and Upt, bilateral, p = 0.0014, p = 0.0027 and p = 0.0028) (Fig. 4B); and (5) the longer and shorter duration groups at the U nerve (Upt, bilateral) (p = 0.0036, p = 0.0005) (Fig. 5B) and the longer duration-control groups at the U nerve (Ut, right and Upt, bilateral, p = 0.0057, p = 0.0005 and p = 0.0001) (Fig. 5B).

The significant differences mainly converged at the M nerve (forearm1/3) and the U nerve (Upt, bilateral), which implied that the M nerve (forearm1/3) and the U nerve (Upt, bilateral) may act as the more valuable positions than the M nerve (forearm1/2) and U nerve (Ut) for detection of nerve enlargement in upper limbs of leprosy patients by US examination.

For the CF nerve, significant differences existed between (1) the leprosy and control groups at the CF nerve (right, p = 0.0264) (Fig. 1C); (2) the MB and control groups at the CF nerve (bilateral, p = 0.0051 and p = 0.0112) (Fig. 2C); (3) the NR and control groups at the CF nerve (right, p = 0.0252) (Fig. 3C); (4) the D and control groups at the CF nerve (right, p = 0.0377) (Fig. 4C); and (5) the longer and shorter duration groups (p = 0.0123 and p = 0.0145) and the longer duration and control groups (p = 0.0115 and p = 0.0036) at the CF nerve (bilateral) (Fig. 5C).

### ΔCSA and ΔUtpt

**Figure 6:**
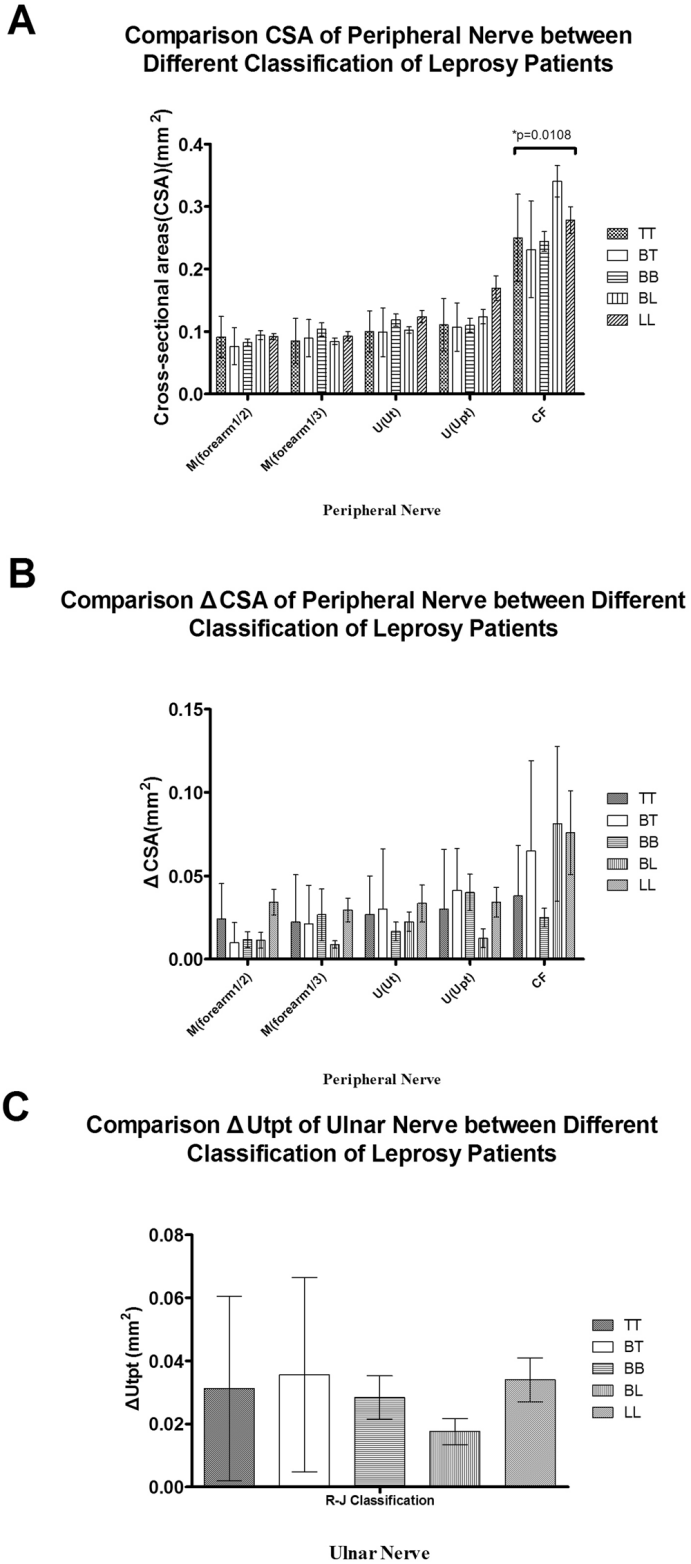
CSA, ΔCSA and ΔUtpt results. The patients were classified according to the Ridley-Jopling classifications. (**A**) CSAs results of the leprosy patients who were classified according to the Ridley-Jopling classifications; (**B**) ΔCSA results of the leprosy patients who were classified according to the Ridley-Jopling classifications; and (**C**) ΔUtpt results of the leprosy patients who were classified according to the Ridley-Jopling classifications. The nonparametric Mann-Whitney U test was used to analyze differences between two groups. The Kruskal-Wallis test was used to analyze differences among three groups. All the statistical analyses were performed using Prism version 5 (GraphPad Software Inc., San Diego, CA, USA). Two-sided p-values < 0.05 were considered statistically significant. *p < 0.05 indicates a significant difference.

We also calculated the CSA, ΔCSA and ΔUtpt according to the Ridley-Jopling classification and the method described in^14^. Only the CSA of the CF nerve exhibited a significant difference between different groups(p = 0.0108), but no significant difference was noted between the ΔCSA and ΔUtpt (Fig. 6). Details of the CSA, ΔCSA and ΔUtpt results are presented in Supplementary Table S3.

## Discussion

Leprous neuropathy, which results from the infection of nerve cells by *M. leprae*, affects millions of people in many developing countries. The clinical and pathological manifestations are determined by the natural resistance of the host to *M. leprae* invasion. Failure of early detection of leprosy often leads to severe disability despite eradication of mycobacteria at a later date^17^.

In this study, we investigated peripheral nerve abnormalities in leprosy patients by US. In the upper limbs (bilateral M and U nerves), the CSAs were generally greater in leprosy patients than those in the controls. In contrast, in the lower limbs (CF nerve, bilateral), the CSAs were reduced in the leprosy patients compared to those in the controls. The same trend was also noted in other groups, such as the MB/PB, R/NR, D/ND and longer/shorter duration groups. Nerve enlargement is generally reported^10,13–15^, whereas neuratrophy is rarely mentioned in leprosy patients. Our study observed that peripheral nerves in the lower limbs (CF) were thinner in almost all subgroups of the leprosy patients than those in the controls, and significantly thinner bilateral CF nerves were noted in the longer duration group than those in the shorter duration group. Additional studies are needed to determine whether neuratrophy is prevalent in leprosy patients after MDT.

In the LL group, bacilli are found in the skin and in nerve cells, including Schwann cells, endothelial cells, and macrophages. In the TT group, a strong cell-mediated immune reaction leads to the formation of granulomas and destruction of cells harboring bacilli and neighboring nerve fibers. Nerve lesions lead to a symmetrical, pseudo-polyneuritic pattern in most cases of LL leprosy, whereas the multifocal pattern is more common in TT leprosy^17^.

For the MB, PB and control groups, CSA values exhibited the following trends: PB > MB > controls in the upper limbs (M/U nerves), and the PB group exhibited a significantly greater CSA than the MB group at the U nerve (Ut, right). Our results differed from another study, which reported that CSA values of the MB group were greater than those of the PB group regardless of pretreatment or post-treatment status and measurement in the upper or lower limbs^16^. In our study, the PB group also exhibited significantly increased CSA values compared to the controls at the M (forearm1/3, left) and U nerves (Ut, right and Upt, bilateral). These findings suggest that nerve enlargement was more common in the upper limbs of the PB group than that in the MB group, especially in the M (forearm1/3, left) and U (Ut, right and Upt, bilateral) nerves. Our study enrolled a larger number of PB patients (n = 34) and focused on different statuses of leprosy patients compared to the previously mentioned study (many of the patients were assessed several decades after treatment), which may explain the differences in the results. According to our study, nerve enlargement is more likely to occur in PB patients than that in MB patients.

For the R, NR and control groups, the CSA values generally exhibited the following trend: R > NR > controls in upper limbs (M/U nerves), except for the M (forearm1/2, right) nerve. Significantly higher values were found in the R vs NR, R vs control, and NR vs control groups at the following positions: the M nerve (forearm1/3, left) and U nerve (Ut, right and Upt, bilateral). This result was similar to another study, which reported that the CSA values of the R group were higher than those of the NR group^16^. The CSA was significantly increased in both the R and NR groups compared to the controls at the M (forearm1/3, left) and U nerve (Ut, right and Upt, bilateral). These findings suggest that nerve thickening is a common phenomenon in the upper limbs of leprosy patients regardless of the presence of a reaction, which may act as a risk factor of nerve enlargement.

Regarding the different disability statuses, the CSA values generally exhibited the following trend: ND > D > controls in the upper limbs (M/U nerves). Significant increases were noted between the ND and control groups at the M nerve (forearm1/3, right) and U nerve (Ut, right and Upt, bilateral) and between the ND and D groups at the U nerve (Ut and Upt, right). These findings suggest that leprosy patients without disability are more likely to exhibit nerve enlargement in peripheral nerves (M/U nerves) in the upper limbs.

Moreover, regarding the CSAs of patients with different durations after treatment, almost all the CSA values presented the following trend in the upper limbs (M/U nerves): longer duration > shorter duration > controls. Significant increases were found in the longer duration group compared to those in the control groups at the following positions: the M nerve (forearm1/3, left) and the U nerve (Ut, right and Upt, bilateral). Significant increases were found in the longer duration group compared to those in the shorter duration group at the U nerve (Upt, bilateral). These findings suggest that neural involvement may not improve after treatment. As time progressed, nerve thickness was more obvious in the upper limbs of leprosy patients with longer duration after treatment.

As we previously mentioned, although the peripheral nerves of the upper limbs of leprosy patients were thicker than those of the control groups, the peripheral nerves of the lower limbs (CF nerve) were thinner in the leprosy patients than those in the control group. Significantly thinner nerves were noted in the MB and longer duration groups at the CF nerve (bilateral) and in the NR and D groups at the CF nerve (right). These findings suggest that nerve enlargement typically occurs in the peripheral nerves of the upper limbs in leprosy patients, whereas neuratrophy may occur in the peripheral nerves of the lower limbs (CF nerve). Whether neuratrophy can be considered a pathological result and/or common occurrence in leprosy patients requires further observations and studies for confirmation. The above data suggest that the most frequently affected nerve was the U nerve, especially at the Ut, right and Upt, bilateral positions, and the most frequently affected side was the right side.

No significant differences in ΔCSA were noted, and no asymmetric nerve enlargement was identified in our study. In addition, no significant differences were identified in ΔUtpt. According to our data, the main significant differences were noted in the U nerve (Ut) at the right side. The bilateral Upt position may represent a more valuable position for nerve enlargement detection than the Ut position.

## Conclusion

In conclusion, a moderate correlation was observed between clinical information (leprosy/control, MB/PB, R/NR, D/ND, and longer/shorter duration groups) and US examination results. The coexistence of nerve enlargement and neuratrophy in the peripheral nerves of leprosy patients represents the most prevalent type of neural impairment. The PB type, presence of a leprosy reaction, lack of disability, and longer duration serve as risk factors for neurologic enlargement in nerves of the upper limbs (M and U nerves) of leprosy patients, whereas the MB type, presence of disability and longer duration may suggest neuratrophy in nerves of the lower limbs (CF nerve). No asymmetric nerve enlargement was identified in our study. The most frequently affected nerve was the U nerve, and the Upt position may represent a valuable position for the detection of nerve enlargement.

## Methods

### Ethics Statement

This study was approved by the Medical Ethics Committee of Beijing Friendship Hospital, Capital Medical University, Beijing, P.R. China. Written informed consent was obtained from all the participants.

### Subjects

Seventy-one leprosy patients who were referred to the Prevention and Control Center of Leprosy in Sanya city, Hainan province, were included in the study and underwent bilateral high-resolution US of the peripheral nerves during MDT as recommended by the World Health Organization (WHO) (n = 1) or after MDT (n = 70). Leprosy diagnosis was established based on the clinical signs and symptoms, skin smears, skin biopsy, and neuro-physiologic examinations when necessary.

### Grouping

The leprosy patients were classified into five groups based on the Ridley and Jopling^18^ classification: the tuberculoid (TT), borderline-tuberculoid (BT), borderline-borderline (BB), borderline-lepromatous (BL), and lepromatous (LL) groups. The patients were also classified as PB and MB according to the WHO operational classification^19^. Medical information was collected for the identification of any leprosy reactions, disability and treatment duration prior to US evaluation. The leprosy patients were classified into R, including Type 1 reactions (cutaneous reactions) and Type 2 reactions (erythema nodosum leprosum) and NR groups; D and ND groups; and longer (>20 years) and shorter duration (<20 years) after treatment groups. For statistical analysis, the patients were divided based on their clinical characteristics.

### US

Clinicians experienced in US performed all the US sessions using a 13–6 MHz linear transducer (M-turbo & S-series, Sonosite, Shanghai, China). The bilateral U, M and CF nerves were systematically scanned along the transverse axes. The M nerves were scanned along the middle part (forearm1/2) and lower third of the forearm (forearm1/3). U nerves were scanned at the Ut and Upt. The patients were examined in a supine or prone position to assess the peripheral nerves in the upper (M/U nerves) or lower limbs (CF nerve). In nine cases, it was not possible to examine the CF nerves bilaterally due to amputation (six in the left leg and three in the right leg).

Nerve CSAs were measured by freehand delimitation at the outer borders of the echogenic rims of the nerves. The measurements were performed using an electronic cursor, and the CSAs were assessed at the level of maximum nerve thickening. The ΔCSA (absolute difference between right and left CSAs) and ΔUtpt (absolute difference between the Upt and Ut CSAs) were also calculated according to a previously described method^14^.

### Statistical Analysis

The statistical analysis was performed using GraphPad Prism software version 5.0 (GraphPad Software Inc., San Diego, CA, USA). The nonparametric Mann-Whitney U test was used to compare the means of two groups. The Kruskal-Wallis test was used to analyze the differences among the means of three or more groups. Probability (p) values less than 0.05 were considered significant.

### Ethical Approval

All the procedures in the studies involving human participants were performed in accordance with the ethical standards of the institutional and/or national research committee and with the 1964 Helsinki declaration and its later amendments or comparable ethical standards.

## Electronic supplementary material

Supplementary Tables 1–3
