# Supplementary material for: Coexistence of Nerve Enlargement and Neuratrophy Detected by Ultrasonography in Leprosy Patients
Source: Sci Rep. 2018 May 17;8:7812. doi: 10.1038/s41598-018-26085-1 (PMC5958074; doi:10.1038/s41598-018-26085-1)
Supplement: Supplementary file 1 — Supplementary Tables 1–3 [file 41598_2018_26085_MOESM1_ESM.pdf]

Coexistence of Nerve Enlargement and Neuratrophy Detected by Ultrasonography in Leprosy Patients

Xiaohua Chen[1,2], Liangfu Zhang[2,3], Meiying Huang[2,3], Xiuli Zhai[2,3],Yan Wen[1,2*], Chunzhi Pan[2,4]

[1] Beijing Tropical Medicine Research Institute, Beijing Friendship Hospital, Capital Medical University, Beijing 100050, China

[2] Beijing Key Laboratory for Research on Prevention and Treatment of Tropical Diseases, Capital Medical University, Beijing 100050, China

[3] Sanya Leprosy Prevention and Control Center, Sanya, Hainan 572000, China

[4] China Leprosy Association, Beijing 100068, China

*** Corresponding author**

Yan Wen

Tel.: +86-10-83168482

E-mail: weny8@163.com

Nerves		CSA	Leprosy	Controls	Classification		Reaction		Disability		Duration	
					MB	PB	R	NR	D	ND	Longer	Shorter
M (forearm1/2)	left	mean(mm2)	0.092	0.084	0.091	0.094	0.098	0.091	0.093	0.091	0.093	0.090
		SD(mm2)	0.031	0.021	0.032	0.031	0.029	0.032	0.029	0.038	0.034	0.021
		n	71	29	34	37	8	63	51	20	53	18
		95% CI	0.085-0.100	0.076-0.092	0.079-0.102	0.083-0.104	0.073-0.122	0.083-0.099	0.084-0.101	0.073-0.109	0.083-0.102	0.079-0.101
	right	mean(mm2)	0.086	0.081	0.084	0.088	0.078	0.087	0.086	0.087	0.083	0.096
		SD(mm2)	0.031	0.020	0.033	0.030	0.024	0.032	0.030	0.036	0.031	0.032
		n	71	29	34	37	8	63	51	20	53	18
		95% CI	0.079-0.094	0.074-0.089	0.072-0.095	0.078-0.098	0.058-0.097	0.079-0.095	0.078-0.094	0.070-0.103	0.075-0.091	0.079-0.112
M (forearm1/3)	left	mean(mm2)	0.0906	0.0697	0.0835	0.0970	0.1275	0.0859	0.0837	0.1080	0.0945	0.0789
		SD(mm2)	0.0414	0.0186	0.0285	0.0500	0.0891	0.0290	0.0261	0.0639	0.0458	0.0208
		n	71	29	34	37	8	63	51	20	53	18
		95% CI	0.081-0.100	0.063-0.077	0.074-0.093	0.080-0.114	0.053-0.202	0.079-0.093	0.076-0.091	0.078-0.138	0.082-0.107	0.069-0.089
	right	mean(mm2)	0.0883	0.0724	0.0885	0.0881	0.1125	0.0852	0.0837	0.1000	0.0896	0.0844
		SD(mm2)	0.0373	0.0172	0.0395	0.0357	0.0618	0.0324	0.0338	0.0438	0.0386	0.0336
		n	71	29	34	37	8	63	51	20	53	18
		95% CI	0.079-0.097	0.066-0.079	0.075-0.102	0.076-0.100	0.061-0.164	0.077-0.093	0.074-0.093	0.080-0.121	0.079-0.100	0.068-0.101
U(Ut)	left	mean(mm2)	0.107	0.099	0.099	0.114	0.158	0.101	0.103	0.118	0.112	0.093
		SD(mm2)	0.045	0.030	0.032	0.054	0.092	0.031	0.037	0.060	0.049	0.024
		n	71	29	34	37	8	63	51	20	53	18
		95% CI	0.097-0.118	0.088-0.111	0.088-0.111	0.096-0.132	0.081-0.234	0.093-0.109	0.093-0.114	0.089-0.146	0.098-0.126	0.082-0.105
	right	mean(mm2)	0.111	0.089	0.099	0.122	0.176	0.103	0.102	0.136	0.117	0.094
		SD(mm2)	0.049	0.023	0.037	0.057	0.102	0.031	0.033	0.073	0.055	0.020
		n	71	29	34	37	8	63	51	20	53	18
		95% CI	0.100-0.123	0.081-0.098	0.086-0.112	0.103-0.141	0.091-0.262	0.095-0.111	0.092-0.111	0.102-0.170	0.102-0.132	0.084-0.104
U(Upt)	left	mean(mm2)	0.132	0.091	0.109	0.153	0.263	0.115	0.122	0.158	0.142	0.102
		SD(mm2)	0.095	0.021	0.041	0.123	0.217	0.050	0.080	0.124	0.103	0.061
		n	71	29	34	37	8	63	51	20	53	18
		95% CI	0.109-0.155	0.083-0.099	0.095-0.111	0.112-0.194	0.081-0.444	0.103-0.128	0.099-0.144	0.100-0.216	0.114-0.171	0.071-0.132

U(Upt)	right	mean(mm2)	0.129	0.091	0.111	0.147	0.254	0.114	0.116	0.163	0.143	0.090
		SD(mm2)	0.082	0.024	0.041	0.104	0.180	0.041	0.059	0.118	0.090	0.025
		n	71	29	34	37	8	63	51	20	53	18
		95% CI	0.110-0.149	0.082-0.101	0.096-0.125	0.112-0.181	0.104-0.404	0.103-0.124	0.100-0.133	0.107-0.218	0.118-0.168	0.078-0.102
CF	left	mean(mm2)	0.279	0.303	0.249	0.301	0.311	0.274	0.283	0.269	0.256	0.347
		SD(mm2)	0.114	0.076	0.071	0.135	0.114	0.115	0.117	0.110	0.091	0.151
		n	65	29	28	37	8	57	45	20	49	16
		95% CI	0.250-0.307	0.275-0.332	0.222-0.277	0.256-0.346	0.216-0.406	0.244-0.305	0.248-0.318	0.217-0.320	0.230-0.282	0.266-0.428
	right	mean(mm2)	0.258	0.299	0.243	0.272	0.314	0.251	0.254	0.269	0.246	0.297
		SD(mm2)	0.098	0.073	0.069	0.118	0.194	0.077	0.076	0.139	0.104	0.065
		n	68	29	32	36	8	60	48	20	52	16
		95% CI	0.234-0.282	0.271-0.327	0.218-0.268	0.232-0.312	0.151-0.476	0.231-0.271	0.232-0.276	0.203-0.334	0.217-0.275	0.263-0.331

Supplementary Table S1. The results of CSAs of peripheral nerves in different groups showes as mean,SD,n and 95% confidence internal.Patients were classified according to the clinic characters, involving leprosy/controls,WHO classification,reaction,disability and duration.n: number of nerves;SD: standard deviation; CI:confidence interval. M:median;U:ulnar;CF:common febular;R:reaction;NR:no-reaction;D:disability;ND:no-disability.

			Classification				Reaction				Disability				Duration			
Nerves	P-value	Leprosy-controls	total	MB-PB	MB-Controls	PB-Controls	Total	R-NR	R-controls	NR-controls	Total	D-ND	D-Controls	ND-Controls	Total	Longer-shorter	Shorter-controls	Longer-controls
M (forearm1/2)	total	0.5131	0.6220				0.6034				0.5753				0.6588			
	left	0.3720	0.5686	0.6387	0.7014	0.2479	0.5537	0.5962	0.2703	0.4631	0.3754	0.4361	0.2290	0.9672	0.4522	0.1886	0.2735	0.8812
	right	0.7559	0.6307	0.3838	0.8453	0.4713	0.6328	0.4044	0.4986	0.6165	0.7892	0.6799	0.6525	0.9424	0.8633	0.9881	0.1951	0.6807
M (forearm1/3)	total	0.0052*	0.0130				0.0060				0.0013*				0.0106*			
	left	0.0045*	0.0078	0.2302	0.0741	0.0016*	0.0040	0.0777	0.0048*	0.0124*	0.0037*	0.0818	0.0279*	0.0017*	0.0058*	0.1114	0.1964	0.0023*
	right	0.0394*	0.1187	0.9908	0.0907	0.0547	0.0816	0.3196	0.1196	0.0544	0.0132*	0.1276	0.1804	0.0051*	0.1050	0.5903	0.2130	0.0378*
U (Ut)	total	0.1166	0.0352*				0.0187*				0.0325*				0.0543			
	left	0.7368	0.5261	0.2951	0.7973	0.4123	0.2114	0.0867	0.1112	0.9966	0.9285	0.3964	0.9398	0.4609	0.3347	0.1650	0.4325	0.4357
	right	0.0158*	0.0043*	0.0464*	0.4830	0.0006*	0.0052*	0.0207*	0.0066*	0.0406*	0.0024*	0.0298*	0.1079	0.0014*	0.0128*	0.0816	0.4779	0.0057*
U (Upt)	total	0.0022*	0.0019*				<0.0001*				0.0011*				<0.0001*			
	left	0.0103*	0.0213*	0.2473	0.0618	0.0089*	0.0047*	0.0307*	0.0057*	0.0274*	0.0114*	0.0936	0.0571	0.0027*	0.0003*	0.0036*	0.6818	0.0005*
	right	0.0050*	0.0046*	0.1317	0.1146	0.0010*	0.0004*	0.0037*	0.0006*	0.0201*	0.0036*	0.0763	0.0257*	0.0028*	<0.0001	0.0005*	0.6257	0.0001*
CF	total	0.0325*	0.0193*				0.0910				0.1175				0.0003*			
	left	0.0905	0.0301*	0.0755	0.0051*	0.6884	0.1629	0.3477	0.7107	0.0730	0.2356	0.6084	0.1231	0.1723	0.0044*	0.0066*	0.3796	0.0115*
	right	0.0264*	0.0522	0.3927	0.0112*	0.1634	0.0833	0.9469	0.3461	0.0252*	0.0842	0.6861	0.0377*	0.0889	0.0024*	0.0084*	0.7486	0.0036*

Supplementary Table S2. The results of statistical analysis of CSAs of peripheral nerves in different groups showes as p value.Patients were classified according to the clinic characters, involving leprosy-controls,WHO classification,reaction,disability and treatment duration.M:median;U:ulnar;CF:common febular;R:reaction;NR:no-reaction;D:disability;ND:no-disability.p-value by Kruskal-Wallis test and Mann-Whitney U test.* Statistically significant.

		Group(n)	TT	BT	BB	BL	LL	p-value
Variable	Nerve	Patients	26	8	6	8	23	
CSA (mm2)	M(forearm1/2)	n	52	16	12	16	46	0.1847
		mean	0.091	0.076	0.083	0.094	0.092	
		SD	0.033	0.030	0.019	0.029	0.033	
		95% CI	0.081-0.100	0.060-0.092	0.070-0.095	0.079-0.110	0.082-0.102	
		abnormal	9	2	1	1	6	
	M (forearm1/3)	n	52	16	12	16	46	0.4215
		mean	0.085	0.089	0.103	0.084	0.093	
		SD	0.036	0.030	0.037	0.021	0.050	
		95% CI	0.075-0.095	0.074-0.105	0.080-0.127	0.073-0.095	0.078-0.108	
		abnormal	10	3	5	4	12	
	U(Ut)	n	52	16	12	16	46	0.2040
		mean	0.100	0.099	0.118	0.103	0.124	
		SD	0.033	0.039	0.035	0.021	0.066	
		95% CI	0.090-0.109	0.078-0.120	0.096-0.140	0.091-0.114	0.104-0.143	
		abnormal	1	1	2	1	6	
	U(Upt)	n	52	16	12	16	46	0.1486
		mean	0.111	0.107	0.110	0.124	0.169	
		SD	0.042	0.039	0.039	0.047	0.136	
		95% CI	0.099-0.122	0.086-0.128	0.085-0.135	0.099-0.149	0.129-0.210	
		abnormal	10	4	1	6	14	
	CF	n	44	16	12	16	45	0.0108*
		mean	0.250	0.231	0.244	0.341	0.278	
		SD	0.070	0.077	0.056	0.100	0.143	
		95% CI	0.231-0.271	0.190-0.273	0.209-0.279	0.287-0.394	0.235-0.322	
		abnormal	0	0	0	0	3	
ΔCSA (mm2)	M(forearm1/2)	n	26	8	6	8	23	0.0968
		mean	0.024	0.010	0.012	0.011	0.034	
		SD	0.021	0.012	0.012	0.014	0.037	
		95% CI	0.016-0.033	0.000-0.020	-0.001-0.024	0.000-0.023	0.019-0.050	
		abnormal	9	0	0	1	6	

ΔCSA (mm2)	M (forearm1/3)	n	26	8	6	8	23	0.3725
		mean	0.022	0.021	0.027	0.009	0.030	
		SD	0.028	0.023	0.038	0.006	0.034	
		95% CI	0.011-0.034	0.002-0.040	-0.013-0.066	0.003-0.014	0.015-0.044	
		abnormal	4	3	0	0	4	
	U(Ut)	n	26	8	6	8	23	0.9545
		mean	0.027	0.030	0.017	0.023	0.033	
		SD	0.023	0.036	0.014	0.017	0.053	
		95% CI	0.018-0.036	0.000-0.060	0.002-0.031	0.009-0.036	0.011-0.056	
		abnormal	3	2	0	0	3	
	U(Upt)	n	26	8	6	8	23	0.0939
		mean	0.030	0.041	0.040	0.013	0.034	
		SD	0.036	0.025	0.027	0.016	0.043	
		95% CI	0.016-0.045	0.020-0.062	0.012-0.068	0.001-0.026	0.016-0.053	
		abnormal	4	3	0	0	3	
	CF	n	19	8	6	8	22	0.4916
		mean	0.038	0.065	0.025	0.081	0.076	
		SD	0.030	0.054	0.014	0.131	0.118	
		95% CI	0.023-0.053	0.020-0.110	0.011-0.039	-0.028-0.191	0.024-0.128	
		abnormal	1	3	0	0	7	
ΔUtpt	U(Ut and Upt)	n	52	16	12	16	46	0.4560
		mean	0.031	0.036	0.028	0.018	0.034	
		SD	0.029	0.031	0.024	0.017	0.047	
		95% CI	0.023-0.039	0.019-0.052	0.013-0.043	0.009-0.026	0.020-0.048	
		abnormal	5	3	1	0	4	

Supplementary Table S3. CSAs, ΔCSAs and ΔUtpt results. Patients were classified according to the Ridley-Jopling classification. n: number of nerves;SD: standard deviation; CI:confidence interval. M:median;U:ulnar;CF:common febular.* Statistically significant.
